# Development of a Methodological Quality Criteria List for Observational Studies: The Observational Study Quality Evaluation

**DOI:** 10.3389/frma.2021.675071

**Published:** 2021-07-14

**Authors:** Marjan Drukker, Irene Weltens, Carmen F. M. van Hooijdonk, Emma Vandenberk, Maarten Bak

**Affiliations:** ^1^ School for Mental Health and Neuroscience (MHeNS), Maastricht University, Maastricht, Netherlands; ^2^ Mondriaan, Maastricht, Netherlands; ^3^ Rivierduinen, Institute for Mental Health Care, Leiden, Netherlands

**Keywords:** observational studies, risk of bias, methodological quality criteria list, cohort study, case-control study, cross-sectional study

## Abstract

**Background:** Existing study quality and risk of bias lists for observational studies have important disadvantages. For this reason, a comprehensive widely applicable quality assessment tool for observational studies was developed.

**Methods:** Criteria from three quality lists were merged into a new quality assessment tool: the observational study quality evaluation (OSQE). OSQE consists of a cohort, case–control, and cross-sectional version.

**Results:** The OSQE cohort, the OSQE case–control, and the OSQE cross-sectional version include all items applicable to that type of study, for example, the representativeness of the study population, the validity of the independent and dependent variables, and the statistical methods used. Before scoring the OSQE, the rater is asked to define how to score items, in detail. A study can obtain a star for each item. Each item also has a veto cell. This cell can be checked when poor quality with respect to that specific item results in a low quality of the study despite stars on other items. Although stars add to a sum score, the comment field is the most important part of the OSQE.

**Conclusion:** The OSQE presented in the current article provides a short, comprehensive, and widely applicable list to assess study quality and therewith risk of bias.

## Introduction

In medicine, psychology, or health sciences, when performing a meta-analysis or systematic review, judgment of the methodological quality of the included studies is essential. For randomized controlled trials (RCTs) (Higgins et al., 2011; De Brouwer et al., 2012) and systematic reviews (Shea et al., 2007; Shea et al., 2009), various quality criteria lists are available. Criteria lists for observational studies are also available (Vandenbroucke et al., 2007; Salzmann-Erikson and Dahlén, 2017; Wells et al., unknown; Ma et al., 2020), but they have disadvantages. The most recent one, that is, the risk of bias in non-randomized studies of interventions (ROBINS-I), is extensive and is based on analogy with RCTs. This makes the instrument more difficult to score and not suitable for all observational studies (Sterne et al., 2016). Accordingly, besides the ROBINS-I, a new, shorter, intuitively understandable, and more comprehensive quality assessment tool is needed to compare the quality of observational studies.

The increase in the number of published studies in the last decades exceeds the ability of researchers and clinicians to keep track of all the new expanding information. That other authors perform systematic reviews and meta-analyses to summarize findings of individual studies is valuable in acquiring and sharing knowledge. With respect to the hierarchy of the level of evidence, RCTs are at the top, and thus, this study design is seen as the gold standard (Grootendorst et al., 2010). Internal validity is high; confounders are avoided by pre-stratification, randomization, and additional methods to create equal groups; and intention-to-treat analysis is performed (Grootendorst et al., 2010; De Brouwer et al., 2012). However, RCTs are not always appropriate, adequate, or possible (Black, 1996; Grootendorst et al., 2010). To increase internal validity, inclusion and exclusion criteria of an RCT are usually so strict that results are valid for a homogeneous subgroup of patients only (Grootendorst et al., 2010). Results could be extrapolated to other patient groups such as patients with comorbidities, drug use, and different age-groups, but it can be doubted whether this is valid. In addition, randomization is not always ethical. Risk factors such as exposure to asbestos cannot be studied in an RCT, neither can patients be forced to refrain from regular treatment when this treatment is proven effective. Furthermore, because the sample size in RCTs is usually low and follow-up is short, rare side effects can only be detected after introduction of a new drug by performing observational studies (Vandenbroucke, 2004). Finally, the number of hypothesized risk and protective factors for a wide variety of diseases and symptomatology is increasing. By first performing observational studies, researchers can identify which factors are most promising to study in an RCT. Thus, observational studies give additional information next to results from RCTs. Thus, when systematic reviews and meta-analyses are performed to integrate results of individual studies, they should also include observational studies. Observational study designs need their own criteria lists to assess methodological quality.

Within the group of observational study designs, the three most important are cohort study, case–control study, and cross-sectional study. A cohort study assesses risk factors in a group of subjects at the baseline and follows this cohort over time to assess the outcome (usually incidence of a disease or mortality). A case–control study selects a group of subjects with an illness (cases) and matches these with healthy controls. Subsequently, risk factors are retrospectively assessed in both groups, in order to analyze what risk factors are associated with the case–control status. A cross-sectional study assesses risk factors and the outcome at the same moment in time. This type of study design can be used to assess associations (e.g., exposure to specific risk factors may correlate with particular outcomes). However, making causal inferences is impossible. More details on epidemiological study designs can be found in epidemiological textbooks [such as Rothman 2018 (Rothman and Lash, 2018)]. This vast amount of potential articles holding valuable information for systematic reviews and meta-analyses needs assessment of methodological quality. Previously, various terminologies have been used. Synonyms such as “methodological validity,” “study quality,” and “methodological quality” have been fashionable at the end of the 20th century and the beginning of the 21st century. Currently, the term "risk of bias" is the standard term used by Cochrane (Higgins et al., 2011). In fact, methodological validity, study quality, and risk of bias are very similar concepts. In the present article, the term methodological quality is used for this construct. When referring to observational studies, some scientists [e.g., (Sterne et al., 2016; Schünemann et al., 2019)] use the term non-randomized studies (NRSs). However, NRSs also include case studies and case reports. The present article addresses study quality of cohort, case–control, and cross-sectional studies only. Case studies and case reports have a different criteria list (Vandenbroucke, 2002; Albrecht et al., 2009). In the present article, the term “observational studies” includes cohort, case–control, and cross-sectional studies, while the term “NRS” is an umbrella term for observational studies, case series, and case reports.

Contemporary with the present article, two systematic reviews were performed, for which a suitable methodological quality list was needed. The first systematic review was assessing factors influencing the development of aggression in psychiatric inpatient units (Weltens, submitted). A search was performed to find studies analyzing factors important for the development of aggression on the inpatient ward, divided in patient, staff, and ward factors. The search yielded mainly cohort and case–control studies. The second systematic review assessed dopamine functioning in populations with an increased risk of developing psychosis (Van Hooijdonk, in prep). The search contained studies that investigated different parts of the dopaminergic system in high-risk populations and yielded mainly case–control and cross-sectional studies.

While scientific researchers perform systematic reviews and meta-analyses, medical and paramedical students, and residents learn to make a “critical appraisal of a topic” (CAT) (De Brouwer et al., 2012). A CAT is almost similar to a systematic review but has certain specific characteristics. In a CAT, the student starts with a question based on a single patient from his own case load and tries to answer this by searching scientific articles as is done in a systematic review. Findings are used for treatment of one specific patient. This knowledge is without doubt necessary throughout the working life of any medical doctor or paramedical professional. Assessment of study quality of observational studies is important not only in systematic reviews but also in CATs. In particular, for CATs, study quality lists should be short and easy to understand.

Several criteria lists for observational studies are available (Ma et al., 2020). However, because they could not be used for the abovementioned systematic reviews, the need for a new methodological quality list becomes imperative. The Newcastle–Ottawa Scale (NOS) (Wells et al., unknown) is most widely used [e.g., (Banning et al., 2019; Henderson et al., 2019)]. However, the NOS has various disadvantages. First, the NOS has a list for cohort and for case–control studies, but not for cross-sectional studies. Second, lay raters are lost in the staccato terminology and layout. Third, the NOS is based on old cohort studies following a group of exposed subjects and a group of nonexposed subjects, while recent cohort studies usually assess multiple exposures in one population (Rothman and Lash, 2018). So, the NOS is outdated when scoring recent cohort studies. In addition, the NOS has never been published in a peer-reviewed journal. For this reason, the date of origin is unknown. The first systematic review using the NOS was published in 2003 in PubMed (Deeks et al., 2003). Finally, an article criticizing the NOS pointed at some limitations, mainly in the case–control criteria, which could be easily solved (Stang, 2010). For example, items for independent case-ascertainment by two reviewers and blind assessment of exposure are included in the NOS, while validity of case ascertainment and exposure assessment in general are more important. In the Results section of the present article, these issues are addressed.

Strengthening the Reporting of Observational Studies in Epidemiology (Strobe) is a 22-item checklist designed for authors of observational studies to improve the quality and generalizability of observational research (Vandenbroucke et al., 2007). It is not designed as a methodological quality list. Because there is no consensus on what criteria list to use, the Strobe is used as a methodological criteria list (Stanton et al., 2016; Umer et al., 2017; Lee et al., 2018). The main disadvantage of the Strobe is that all items prescribe where in the text information should be provided. This increases readability of the articles, but not all items are applicable for methodological quality. In addition, even items that are related to methodological quality aim at reporting rather than methodological quality (e.g., “Clearly define all exposures,” while “Is the assessment of the main independent variable valid?” would score quality). Therefore, the Strobe is not deemed efficient to assess the quality of observational studies.

Recently, a new criteria list for all NRSs regardless of the study design was developed, the ROBINS-I (Sterne et al., 2016). After three years of expert meetings and feedback, the final instrument was ready. ROBINS-I includes several domains, and each domain starts with a signaling question. Although the ROBINS-I is designed for NRSs, the rater starts with a “target RCT” studying the same research question. Bias is the expected difference between the hypothetically performed target RCT and the NRS of interest. Therefore, the terminology used, the description, and elaboration of the bias, are as if judging RCTs. For example, the term “intervention” actually means “exposure” (Sterne et al., 2016). Other disadvantages are as follows: (a) although developed for all study designs, the ROBINS-I is especially useful for studies with cohort-like designs. It is likely that modifications are desirable for other study types (Sterne et al., 2016). (b) As the ROBINS-I is very detailed, using it is very complicated and time-consuming. (c) Well-known fallacies and flaws of several study designs (Rothman and Lash, 2018) are not included, such as exposure to the independent variable, exclusion of subjects where the outcome is present at the baseline, and length of follow-up. In Discussion, more details are provided.

The abovementioned disadvantages of the NOS, Strobe, and ROBINS-I prevented us from using one of these quality lists for our planned meta-analyses. Earlier, 80 observational study quality lists were found, of which none was identified as the single comprehensive quality assessment list (Sanderson et al., 2007; Dekkers et al., 2019). Because not all raters of observational studies develop their own quality list as suggested in this earlier overview (Dekkers et al., 2019), a short but universal quality list for future meta-analyses, systematic reviews, and CATs using observational studies is needed.

Consequently, the aim of the present study was to compose a comprehensive and widely usable quality criteria list for observational studies: the observational study quality evaluation (OSQE). Two abovementioned criteria lists for observational studies (STROBE and NOS) (Vandenbroucke et al., 2007; Wells et al., unknown) and a criteria list for RCTs (De Brouwer et al., 2012) serve as the basis for the OSQE. In addition, other criteria lists are checked to find additional items. The OSQE intends to compare methodological quality of the studies using the same study design, as opposed to the ROBINS-I. Quality scores can be used to perform sensitivity analyses excluding poor quality studies or can be included as a modifier in meta-regression analysis. The OSQE includes separate quality lists for cohort studies (OSQE cohort), case–control studies (OSQE case–control), and cross-sectional studies (OSQE cross-sectional). The OSQE assesses methodological quality only. For guidelines on how to perform and report a systematic review, the preferred reporting items for systematic reviews and meta-analyses (PRISMA) is recommended (Moher et al., 2009).

## Methods

All items of the NOS and the Strobe (Vandenbroucke et al., 2007; Wells et al., unknown) (observational studies) and non-RCT items of a criteria list for RCTs (De Brouwer et al., 2012) were combined in a new list. The three lists are described below.

The NOS (Wells et al., unknown) consists of two checklists: one for cohort studies and one for case–control studies. They include items on case definition (case–control studies), exposure assessment, and representativeness. The full criteria list can be obtained *via* a Web site (Wells et al., unknown).

The Strobe (Vandenbroucke et al., 2007) consists of 18 items for cohort, case–control, and cross-sectional studies and three items that are specific for one of those three. Introduction, Method, Results, and Discussion sections of an article each have a set of items.

Although an RCT is a different type of research and not all items of an RCT checklist are applicable, checking usefulness of the items of an RCT-criteria list can help when designing a new criteria list. For development of the OSQE, the 10-item criteria list for RCTs used in CAT education in Maastricht was selected (De Brouwer et al., 2012). This list was based on other criteria lists (Badendoch and Heneghan, 2002; Offringa et al., 2008). All items that are applicable to observational studies were selected (e.g., representativeness of the study population, impact of confounders, and loss to follow-up).

When integrating the three abovementioned methodological criteria lists, various stages were completed. First, items from the three lists were combined, and language was improved. Reporting criteria and items only applicable to RCTs were removed. Second, the information sheet was added (see Supplementary Material). Third, an additional file with clear explanation per item was written. Furthermore, all other available methodological quality criteria lists for observational studies were checked for additional items (see *Discussion* of the present article). Subsequently, the OSQE including the additional file was piloted in seven raters; they gave feedback to the epidemiologist. Where needed, the OSQE and explanation file were revised. Finally, two sets of raters rated articles for systematic reviews independently in order to obtain reliability (the Pearson correlation).

## Results

**FIGURE 1:**
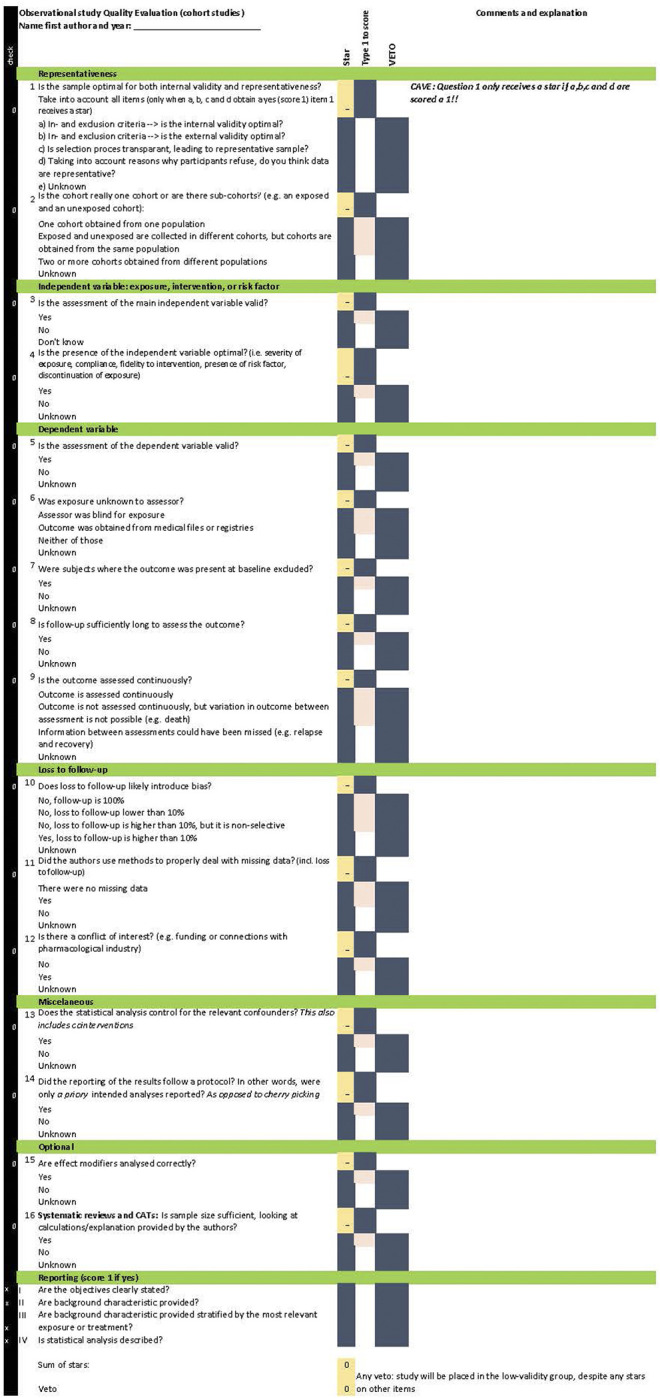
OSQE (cohort).

**FIGURE 2:**
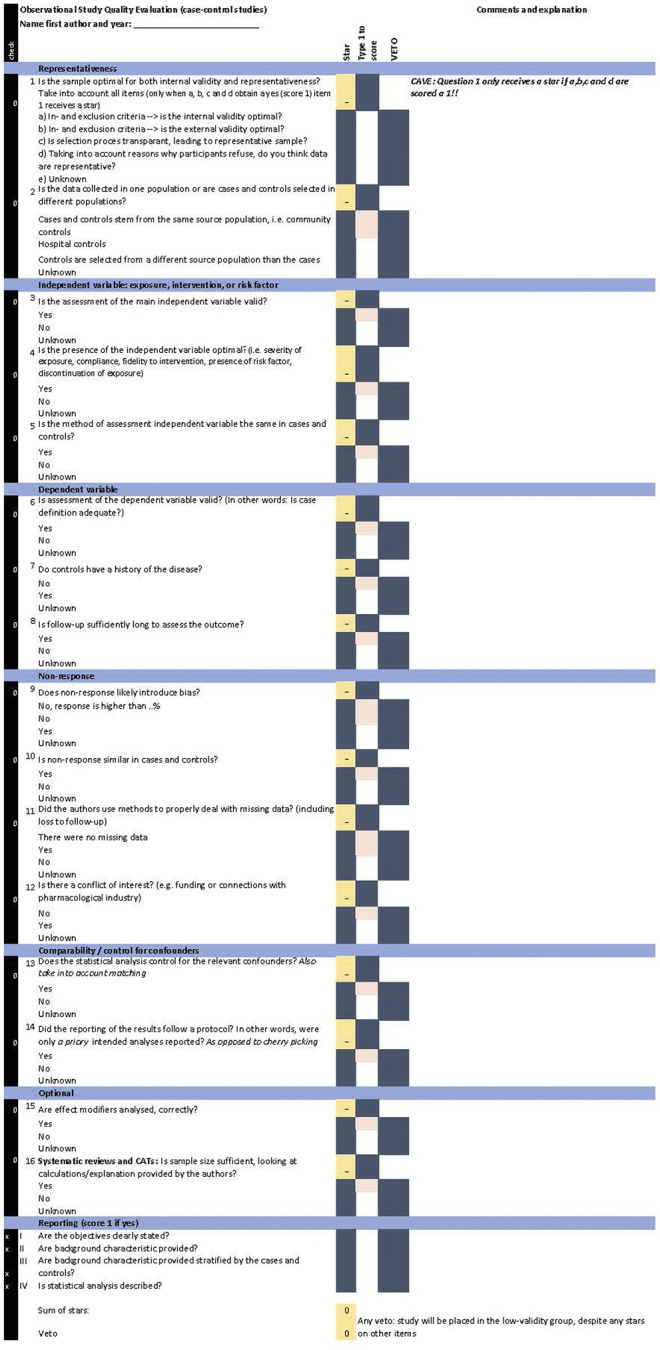
OSQE (case–control).

Figures 1, 2 provide the OSQE cohort and the OSQE case–control, respectively. The OSQE cross-sectional includes a selection of the OSQE cohort items (see below). An Excel file including the OSQE cohort, OSQE case–control, and the OSQE cross-sectional is available in the Supplementary Material. All criteria of all three included criteria lists [10-item criteria list for RCTs, NOS, and Strobe; (Vandenbroucke et al., 2007; De Brouwer et al., 2012; Wells et al., unknown)] were included with a few exceptions. First, criteria prescribing the section of the article where something should be described (reporting criteria; e.g., in the Strobe) were omitted, with the exception of four items at the end of the OSQE. Those four items were not included in the scoring, but could help obtain insights into the quality of reporting. Second, criteria specific for an RCT such as randomization and blinding from the RCT criteria (De Brouwer et al., 2012) were excluded from the pool of items. Finally, one item of the Strobe items (provide study design early in Methods) was extended to reflect a concept that would otherwise be missing (item 14 “Did the reporting of the results follow a protocol? In other words, were only *a* priory intended analyses reported?”). This item is included in other criteria lists, as presented in Discussion (Downs and Black, 1998; Sterne et al., 2016).

The OSQE includes multiple-choice items. For each item, the rater has to add qualitative comments. All items also include the answer “unknown.” Raters check this answer when the answer is not provided in the article or any other article presenting the same study (e.g., an earlier methods article). The OSQE items are rather short so that the rater can add notes. An extra explanation with each item is included in a separate file (Supplementary Material: extra explanation). Analogue to the NOS (Wells et al., unknown), each question receives a star when the most optimal answer is given. It is possible that authors of an original study made a crucial error. In that case, the study quality is poor, despite the number of stars obtained at the other items. Therefore, the OSQE also includes a veto column. Checking the veto column automatically places the article in the low-validity group, despite any stars on other items.

### Information Sheet

Because OSQE items are concise and universal, specification of the items is needed depending on the research question. Thus, before starting the scoring process, the rater needs to define how to score all included studies. For this reason, an information sheet is added to the OSQE (Table 1 and 2, the first Excel sheet in the OSQE Excel file, Supplementary Material). Various items have predefined questions. Raters can add information explaining any other item if needed. When performing a systematic review or meta-analysis, it is recommended that two or more raters reach consensus, also in agreement with PRISMA (Moher et al., 2009). In addition, the information sheet needs to be filled in transparently. When scoring only one or two observational studies (e.g., for a CAT), this process could be more implicit.

**TABLE 1:** Information sheet; several questions to be answered before scoring the OSQE cohort. Information sheet Raters performing a systematic review or meta-analysis: Please answer the questions below before scoring the OSQE. File the marked articles. Answer the questions for your review, not for each article separately. For example, your main dependent variable can be a secondary outcome in the article, but still, you have to score the validity of that outcome. Cave: Numbers correspond with the numbers in the OSQE. For items 1, 3, 4, 5, 8, 10, 13, and 15, explanation is obligatory. Information needed for other items can be added by inserting extra rows in the information sheet.

1	This item of the OSQE searches for a good balance between internal and external validity. Please define which in- and exclusion criteria are OK and which are not. This depends on your choice for this balance in the articles in your meta-analysis.
1	A specific question: What response rate is acceptable? When response rate is low, representativeness is not OK.
3	What are the main independent variables?
4	How is optimal exposure defined?
5	What are the dependent variables of interest?
8	What is the minimum follow-up duration that you think is adequate? (Assuming average follow-up, when follow-up duration varies)
10	Loss-to-follow-up lower than 10% does not introduce bias. For your systematic review, do you think this 10% is the correct cutoff. If not, change.
13	Which confounders are relevant?
15	Are there any hypotheses of effect modification? If yes, what is the effect modifier? Please include question 15 in the scoring.

**TABLE 2:** Information sheet; several questions to be answered before scoring the OSQE case-control.

	For items 1, 3, 4, 6, 7, 8, 9, 13, and 15 explanation is obligatory. Information needed for other items can be added by inserting extra rows.
1	This item of the OSQE searches for a good balance between internal and external validity. Please define which in- and exclusion criteria are OK and which are not. This depends on your choice for this balance in the articles in your meta-analysis
1	A specific question: What response rate is acceptable? When response rate is low, representativeness is not OK.
3	What are the main independent variables?
	How likely is recall bias with this research question?
4	How is optimal exposure defined?
6	What criteria are defined to select cases?
7	Does the control group need to be disease free? (If not omit item 7)
8	What is the minimum duration between exposure and outcome that you think is adequate?
9	Cutoffs for fair, good, and excellent responses could be 60 and 90%. The rater needs to define what response percentage is excellent in this area of research.
13	Which confounders are relevant? The rater has to keep in mind that even matching variables should be controlled for in the analysis.
15	Are there any hypotheses of effect modification? If yes, what is the effect modifier? Please include question 15 in the scoring.

### Cohort Studies

The OSQE cohort is presented in Figure 1 and in the Supplementary Material. The OSQE cohort includes 14 obligatory items. In addition, two items are optional. First, when effect modification is likely in the included original studies, an extra item should be checked (item 15). Second, when raters are not going to perform a meta-analysis, the sample size should be rated (item 16). In a meta-analysis, outcomes of the studies are weighted taking the sample size into account, making this item redundant. Thus, original studies can obtain up to 14, 15, or 16 stars.

### Case–Control Studies

The OSQE case–control (Figure 2 and Supplementary Material) also includes 14 obligatory items and two optional items. Optional items are the same as in the OSQE cohort. Items 2, 6, 7, 9, and 10 are different from those in the OSQE cohort version. These items inquire whether cases and controls stem from the same source population, ascertainment of cases and controls, disease-free controls, response, and differential response between cases and controls.

### Adapt Case–Control List to Meet Earlier Critique

Earlier, the case–control version of the NOS was criticized (Stang, 2010). Below, the critiques are addressed, consecutively. First, the NOS item on case ascertainment in case–control studies is interpreted differently in the OSQE (question 6). In the NOS, case ascertainment by two independent researchers was crucial. Instead, the OSQE asks for validity of case ascertainment in general to be specified by the rater. This way the critique on the NOS is incorporated (Stang, 2010). Second, in the NOS, case–control studies with hospital controls do not obtain a star. This is the same in the OSQE (item 2), while the critique does imply that studies using hospital controls do obtain a star (Stang, 2010). Despite hospital controls stem from the same source population, the use of this type of controls can introduce bias. For example, when patients with a broken leg are selected as hospital controls in a lung cancer study, this may lead to the false conclusion that performing sports protects against lung cancer. In general terminology, the reason why controls are admitted to the hospital seems to protect for being a case (Rothman and Lash, 2018). In addition, when hospital controls are suffering from a disease with the same risk factor as the disease under study, the risk factor is biased toward no association (Rothman and Lash, 2018). Finally, the OSQE judges validity of the assessment of exposure more important than blinding the assessors for case-status (item 3). This is in agreement with the critique (Stang, 2010).

### Cross-Sectional Studies

A subset of the OSQE cohort can be used to score cross-sectional studies, that is, items 1, 3, 4, 5, 12, 13, and 14 (11, 15, and 16 are optional, see Supplementary Material). Items with respect to follow-up and exclusion of subjects at the baseline are not applicable, and thus validity of this study design is intrinsically lower. However, the other items can be scored, enabling comparison of methodological quality within a group of cross-sectional studies. For example, items focusing on representativeness and confounding remain important.

### Reliability

In running meta-analyses, reliability of the OSQE scores was analyzed. The Pearson correlation coefficient of the OSQE cohort and OSQE case–control was *r* = 0.71 (*n* = 45) and *r* = 0.80 (*n* = 8), respectively (Weltens, submitted). In another meta-analysis, the Pearson correlation coefficient of OSQE case–control was *r* = 0.81 (*n* = 21) and *r* = 0.51 (*n* = 11), respectively (two second raters), and the Pearson correlation coefficient of OSQE cross-sectional was *r* = 0.65 (*n* = 7) (Van Hooijdonk, in prep). Neither of the raters used a cutoff point.

## Discussion

The OSQE provides a comprehensive and widely applicable list for the assessment of study quality in observational studies. The OSQE is based on existing quality assessment lists. All items are included, but items that are criticized in the literature have been adapted. Each item has a comments field, and those qualitative comments are most important. However, stars and the sum of stars are included to have a rough tool to discriminate study quality.

### Points of Attention for Raters

Both users of the OSQE and raters of other methodological quality lists should comply with various general guidelines. First, when performing a systematic review or meta-analysis, raters should file the marked articles for reasons of transparency (Dekkers et al., 2019). Second, specification of criteria on how each item should be scored is crucial for every research question. For this, the information sheet is added to the OSQE Excel file (Table 1). Third, initially, none of the eligible studies should be excluded from a meta-analysis because of poor methodological quality. A sensitivity analysis can be performed excluding the poor-quality studies (subgroup analysis). Alternatively, a dichotomous or categorical study quality variable can be added as a modifier to a meta-regression analysis. When multiple observational studies all have the same methodological problem, this flaw can be analyzed separately (the presence or absence of the flaw as a modifier) because it has been shown that this can impact the results (Stroup et al., 2000). Finally, it is advised not to use weights based on methodological quality (Stroup et al., 2000; Dekkers et al., 2019) because when summing the stars, all criteria are considered equally important, while this is not the case. Weighting cannot solve this problem because all weights are arbitrary. Therefore, a general cutoff point for the number of stars to discriminate between good and poor study quality is not provided. For abovementioned sensitivity analysis or inclusion of a moderator, the rater can determine the optimal cutoff point. By including all vetoed studies in the poor-quality category, categorization of the studies better reflects real study quality.

### Other Quality Lists for Observational Studies

There is more literature on study quality/risk of bias than the lists used in the present article. Various articles were scrutinized to improve the OSQE. First, the critical appraisal tools provided by the Joanna Briggs Institute of the University of Adelaide included lists for three types of observational studies: cohort, case–control, and cross-sectional studies (Joanna Briggs Institute, 2017). The Joanna Briggs Institute cohort list included one item that was not included in the OSQE, that is, similarity of exposure assessment in exposed and unexposed subjects. The assumption was that only a minority of the recent cohort studies include a cohort with exposed and another cohort with unexposed subjects. For this reason, this item was not added to the OSQE. Additionally, the Joanna Briggs case–control list included four items that were different from the OSQE: an item on matching, an item on the comparability of the groups, an item on control for confounders, and an item on the appropriateness of the analyses. Because of overlap between those four, the OSQE combined all in one item (item 13). An item whether “the same criteria were used for identification of cases and controls” was not included in the OSQE. This can be scored as part of item 6 (adequate case definition).

Second, “conducting systematic reviews and meta-analyses of observational studies of etiology” (COSMOS-E) provides a set of seven principles to comply with when assessing the quality of observational studies (Dekkers et al., 2019). The OSQE complies with the COSMOS-E principles. For example, principle 4 argues that risk of bias should be assessed per outcome variable, and principle 5 prescribes that the article copies used for the scoring should be filed for transparency. To comply with principle 7, the COSMOS-E tip to think of the perfect study is added to the additional file. Both the COSMOS-E (principle 2) and the OSQE stress the importance of qualitative comments. Despite that, the OSQE does include a sum of stars trying to discriminate between good and poor quality [against principle 6 of the COSMOS-E: “summary score should be avoided” (Dekkers et al., 2019)]. Otherwise, analyzing whether the results are different depending on the quality of the study is not possible. A veto column categorizing a single study as poor quality whatever stars it received on any of the other items partly removes the drawbacks highlighted in the COSMOS-E. Principle 1 suggests a universal criteria list as the OSQE is impossible. Instead, areas to be scored should be selected for each study domain separately (Dekkers et al., 2019). It is unlikely that researchers, medical doctors, and students generate a new quality list for each CAT, systematic review, meta-analysis, or assignment, for reasons of time constraints and limited methodological expertise. To comply with principle 1 of the COSMOS-E, the OSQE includes an information sheet with specific questions per item that the rater should fill in before scoring articles for that specific research question (Table 1) (Dekkers et al., 2019).

Third, the quality index developed by Downs and Black (Downs and Black, 1998) (hereafter D&B) aimed to score both RCT and NRSs using the same instrument. D&B pleas for the use of subscales; the authors argue that authors of NRSs should discuss consequences of weaknesses rather than only generating a sum score. For the same reason, the OSQE encourages qualitative assessment. Both D&B and OSQE include external validity of the study, as opposed to the ROBINS-I. While D&B includes separate items for internal and external validity, the OSQE asks the rater to judge the balance between internal and external validity. This is in agreement with the 10-item criteria list for RCTs (De Brouwer et al., 2012) and with the fact that an increase in internal validity always goes at the expense of external validity and *vice versa* (Rothman and Lash, 2018). As the Strobe, D&B includes reporting items in their quality index sum score. In 1998, techniques for meta-analysis were still in their infancy, and thus, the authors still included power and even suggested to give less weight to null findings in small studies. Nowadays, this would be considered incorrect. Because of publication bias, null findings are often underrepresented (Sharma and Verma, 2019). When performing a meta-analysis, sample size/power does not need to be scored because the meta-analysis generates a result weighted by the number of subjects. The OSQE does include an optional item on sample size/power. As soon as the rater does not perform a meta-analysis, this item should be scored. Except for reporting items, power, and RCT items, all D&B items are also included in the OSQE.

Furthermore, the Critical Appraisal Skills Program (CASP) provided lists for cohort and case–control studies (Critical Appraisal Skill Program, 2018; Ma et al., 2020). The CASP case–control and cohort were designed for medical students. It was better suited for the use in education, than for the use in systematic reviews. The CASP missed a lot of items that were included in other methodological criteria lists (internal validity–external validity, specific flaws for cohort case–control designs, and missing data). The CASP did include various items that were not in the OSQE. However, these do not belong in a methodological quality list (e.g., “What are the results of this study,” “do you believe the results,” and “what are implications of this study for practice?”). In addition, the screening questions were too broad, including multiple topics combined. The National Heart Lung and Blood Institute (NIH) and Scottish Intercollegiate Guidelines Network (SIGN) were rather similar to the OSQE lists, with some minor differences (National Heart Lung and Blood Institute, unknown; Scottish Intercollegiate, unknown).

Finally, the ROBINS-I was developed recently (Sterne et al., 2016) Table 3 presents differences between OSQE and ROBINS-I in more detail. The ROBINS-I intends to harmonize scoring between study designs (RCT, cohort, case–control, cross-sectional, case series, and case reports), while the OSQE aims to assess study quality within studies sharing the same study design (e.g., cohort studies only). When a meta-analysis includes both RCTs and NRS’, ROBINS-I could be a better choice. However, it is also possible to use a different criteria list for each study design and add the variable “study design” as a modifier to the meta-analysis. While the ROBINS-I is extensive and difficult to score for some raters, the OSQE aims to be both comprehensive and comprehensible for raters with various levels of expertise. The OSQE includes some items that the ROBINS-I does not include (Table 3). Another difference is that the OSQE emphasizes the importance of qualitative information, while the ROBINS-I only asks for quantitative scoring (Sterne et al., 2016; Schünemann et al., 2019).

**TABLE 3:** Comparison between ROBINS-I and OSQE. Section A: Overlap between ROBINS-I and OSQE^a^.

ROBINS-I	Bias due to …	OSQE cohort	OSQE case–control
Pre-intervention	.. Confounding	13	13
	.. Selection of participants	1	1
At intervention	.. Classification of interventions	3	3
Post-intervention	.. Deviation from intended interventions	4^b^	4^b^
	.. Missing data	10, 11	9, 11
	.. Measurement of the outcomes	5, 6	6
	.. Selection of the reported results	14	14

a ROBINS-I and OSQE have in common that raters are asked to define several items, before starting to rate, for example, the dependent and independent variables of interest. ROBINS-I additionally asks for the research question and PICO (patient, intervention, comparison, and outcome).

b Similar but not the same.

**Table:** Section B: OSQE items that were not in ROBINS-I.

OSQE cohort		Reason for inclusion in OSQE
2	Inclusion of cohorts from multiple source populations	In older cohort studies this did happen and this could introduce bias.
4	The presence of the independent variable	ROBINS-I inquires the deviation from the intended intervention (even including the balance between intervention groups). OSQE item 4 is broader (all reasons why exposure is lower) and does not inquire the balance between groups (assuming that control groups do not always have an alternative exposure).
7	Exclusion of subjects, where the outcome is present at the baseline	Obviously, in an RCT those subjects are also excluded, but criteria lists for RCTs do not include this criterion. In a cohort study, the likeliness of this is much higher.
8	Follow-up sufficiently long.	When follow-up is too short, the outcome may not have occurred.
9	Continuous assessment of the outcome	As opposed to RCTs, longitudinal observational studies sometimes have no follow-up assessments, but instead use existing registrations and databases.
12	Conflict of interest	In criteria lists for both RCTs and observational studies, there is a debate whether or not to include conflict of interest as a criterion. The OSQE includes the item because it was in the NOS. It has been reported that conflict of interest is associated with bias (Claxton, 2007; Wilson, 2016).
14	Effect modification	When there is a hypothesis for interaction, ignoring this would lead to erroneous results (Rothman and Lash, 2018).
15	Sample size	Because the ROBINS-I is designed for meta-analyses only. Raters using the OSQE are instructed to omit this item, when performing a meta-analysis.
OSQE case–control		
2	Cases and controls stem from different populations	Because of the difficulty to define the source populations of cases, it is not obvious that cases and controls stem from the same population and this can introduce bias. In addition, the use of hospital controls can also introduce bias.
4	The presence of the independent variable	See the OSQE cohort.
5	Assessment of the independent variable that is the same in cases and controls	In case-control studies data collection is often different in cases and controls.
7	Do controls have a history of the disease	When taking a random sample of the healthy population or when matching with the healthy population, a percentage similar to the prevalence of the disease of interest will have the disease.
8	Follow-up sufficiently long	See the OSQE cohort.
10	Nonresponse similar in cases and controls	It is very well likely that those cases are much more motivated to take part in the study than healthy controls who have no interest in the disease of interest.
12	Conflict of interest	See the OSQE cohort.
14	Effect modification	See the OSQE cohort.
15	Sample size	See the OSQE cohort.

An extended elaboration on fallacies in case–control and cohort studies can be found in epidemiology books (Rothman and Lash, 2018).

Other recent methodological quality criteria lists were generated after extended periods of expert meetings or by performing factor analysis (Shea et al., 2007; Sterne et al., 2016). Despite the overlapping items, available methodological quality lists for observational studies were not suitable for the abovementioned systematic reviews (Weltens, submitted, Van Hooijdonk, in prep). For this reason, the OSQE combined existing methodological quality lists, rather than going through the process to generate a new criteria list from scratch. A limitation is that the OSQE is put together by a single epidemiologist. However, exclusion of items was minimal and is transparently explained in the present article. When checking all other existing criteria lists, no other items were found. Extra attention was paid to readability and understandability. For this, the OSQE was piloted. This way, an instrument was created that is suitable for consensus, reliable, and broadly applicable, and also available to be used in our meta-analyses.

## Data Availability

TThe data used for the analyses (reliability) are included in the Supplementary Material.

## Abbreviations

CAT, critical appraisal of a topic; COSMOS-E, conducting systematic reviews and meta-analyses of observational studies of etiology; D&B, Downs and Black; OSQE, observational study quality evaluation; NOS, Newcastle–Ottawa scale; RCT, randomized controlled trail; ROBINS-I, risk of bias in non-randomized studies of intervention assessment tool; Strobe, strengthening the reporting of observational studies in epidemiology.
