# Supplementary material for: Development of a Methodological Quality Criteria List for Observational Studies: The Observational Study Quality Evaluation
Source: Front Res Metr Anal. 2021 Jul 14;6:675071. doi: 10.3389/frma.2021.675071 (PMC8317224; doi:10.3389/frma.2021.675071)
Supplement: Supplementary file 3 [file Table1.docx]

**Additional file: Explanation of each OSQE item**

*On the next pages, items included in the OSQE are further explained. In general, a study can obtain a star on each of the questions. The OSQE has a cohort version, a case-control version and a cross-sectional version. All three versions have a different excel scoring sheet. So, before starting to rate, the raters need to be able to identify the study design^1^. The OSQE cross-sectional is a selection of items from the OSQE cohort. So, for further explanation the rater can read the specific items of the OSQE cohort.*

*For each separate systematic review or meta-analysis, the rater (i.e. the person using the OSQE to rate quality of observational studies) needs to provide a description of several issues in order to be able to score the OSQE in a transparent and consistent way. (When making a CAT, this description is often more implicit and the information sheet does not need to be used). When providing this information, the rater is advised to imagine a study in the domain of the research question without bias (e.g. an RCT). The OSQE excel file includes a separate sheet to write down this extra information (information sheet). For several items, questions are specified. When the rater feels the need to explain other items, these items can be added to the information sheet. To make the information sheet more objective, it is recommended that two or more raters reach consensus.*

*Although the OSQE results in a sum of stars, the comments and explanation field are more important (see main article). Each item also has a veto cell. The rater checks this cell when a specific item leads to so much bias that validity of the article is poor despite stars on any other item. For example, when duration of the trial is two hours, while effects are expected to be visible in a week.*

*To score the OSQE an excel file including formulas can be used (supplement). In this file, answers must be provided by typing a 1 in the corresponding cell. Analogue to the NOS (Wells et al., unknown), each question receives a star when the most optimal answer is given. For example, when the answer to question 3 is yes (pink cell), question 3 receives a star. Question 1 is an exception; this question only receives a star when all items are scored a yes (meaning a good balance between internal and external validity, and a transparent selection process and non-selective drop-out). In all other questions, answers are mutually exclusive.*

*The original excel file includes formula’s (yellow cells). The full question automatically receives a star when a pink cell is checked. When scoring manually, this same principle can be used to provide stars. Formulae’s in the last two rows of the OSQE include a sum of all stars and a veto that is 1 if the veto box was checked once. When testing the OSQE, it turned out that questions are easily omitted. Cells in the check column in black include formulae’s whether the question is filled in (1) or not (0).*

*The rater is obligated to file the marked articles for transparency.*

*Tip: When the rater keeps in mind the perfect unbiased study, this helps in answering the questions on the information sheet and scoring the OSQE. Good examples of unbiased studies are RCT’s; although impossible to perform this study design can still serve as hypothetical unbiased study (Dekkers et al., 2019).*

*Footnote*

*^1^ When piloting the OSQE, raters sometimes filled in the wrong form.*

*Definitions:*

*Author = author of original observational study;*

*Rater = the person who uses the criteria list for a systematic review, meta-analysis or CAT (critical appraisal of a topic; for medical students).*

*Original study = observational studies to be included in the systematic review or CAT.*

*Criteria lists used to generate the OSQE*

*Abbreviation Name in full Reference (see main article)*

NOS Newcastle-Ottawa Scale (Wells et al., unknown)

Strobe Strengthening the Reporting of Observational (Vandenbroucke et al., 2007)
 Studies in Epidemiology

*Criteria lists checked to make sure OSQE does not miss items*

COSMOS-E Conducting Systematic Reviews and Meta-Analyses of (Dekkers et al., 2019)
Observational Studies of Etiology

D&B Downs and Black (Downs and Black, 1998)

NIH National Heart Lung and Blood Institute (National Heart Lung and Blood
 Institute, unknown)

-- Joanna Briggs Institute (Joanna Briggs Institute, 2017)

ROBINS-I Risk Of Bias In Non-randomized Studies – (Sterne et al., 2016)
of Interventions assessment tool

SIGN Scottish Intercollegiate Guidelines Network (Scottish Intercollegiate
 Guidelines Network, unknown)

| **Epidemiology books including a better explanation of the epidemiological background**   - Kenneth J Rothman, Timothy Lash. Modern epidemiology, 4^th^ revised edition, May 2018, Lippincott Williams And Wilkins. ISBN-13: 978-1451193282   Epidemiology in Dutch:   - L.M. Bouter, M.C.J.M van Dongen, G.A. Zielhuis, M.P.A. Zielhuis. Leerboek epidemiologie. 7e druk, april 2016. ISBN 9789036805612 |
| --- |

*A Cohort studies*

1. ***Is the sample optimal for both internal validity and representativeness?***

Criterion 1 judges the correct balance between internal and representativeness (external validity). Per definition, an increase in internal validity leads to a decrease in external validity: internal validity is highest in a homogeneous study population with multiple in- and exclusion criteria. External validity is highest when all subjects from a study population are included in the study. So, the more in- and exclusion criteria are formulated the higher the internal validity at the expense of the external validity. For this reason, both items a and item b need to be optimal in order to gain a star. The rater needs to define what is optimal balance per research question. Thus, a question in the information sheet asks for this balance.

Items c and d further explore representativeness and also need to be optimal. Thus, while on other items a yes on one of the sub-items is sufficient for a star, on this item a star is only given when a to d all are scored yes.

Items c and d: Optimal representativeness means that all subjects of a population are included or a random sample of them. When participants are recruited after referral to a specialist or hospital department, words such as “consecutive” or “all” are informative when the rater wants to know that all patients are selected. This also means that selection is not associated with either exposure or outcome.

When items a to c point at a representative sample, non-response/refusal can still jeopardize representativeness; mainly when non-response rate is high or selective. Item 1d addresses participation / non-response bias, i.e. when non-participating individuals differ from participating individuals with regard to the assessed independent (exposure) and/or dependent variables (outcome). For example, if one selects a sample of 100 nurses and sent them a survey about their workload. The nurses with a high workload may not complete the survey because they have not enough time to complete it. This selective participation can affect the external validity of the study results. Therefore, it is of importance to evaluate whether there are reasons for individuals to not participate in the study.

As a rule of thumb, the rater can assume that a response rate of 80% is good. However, in some areas this is not feasible. In the information sheet, the rater can define what is a good response for the specific research question.

Favorable answers:

1a: optimal internal validity

1b: optimal external validity

1c: transparent selection process and representative sample

1d: no reasons for refusal that point at non-representative data

1. ***Is the cohort really one cohort or are there sub-cohorts, e.g. an exposed and an unexposed?***

In earlier studies, exposed and unexposed were often derived from different populations. Differences between the populations can lead to biased results. Thus, a study obtains a star when all subjects are collected from the same population. When a study oversamples a subgroup within a population to include in either exposed or unexposed this also need to be scored as c (2 or more cohorts from different populations, no star).

1. ***Independent variable: validity of assessment***

What instruments were used to assess the independent variables (exposure) as explained in the information excel sheet? The answer “yes” can be filled in when validity of all instruments is judged as good. When assessments of some exposures are valid and assessments of others are not, the comment field can be used to specify.

1. ***Presence of exposure.***

When subjects are not compliant to medication in a randomized controlled trial (RCT), exposure is not as high as intended. Analogues with that, exposure in observational studies can also differ. For example, (1) also in cohort studies, subjects can decide not to take prescribed medication; (2) Exposure to pollen (hay fever) will be far lower when it rains throughout the study period. It depends on the research question how likely differences in quantity of exposure are.

1. ***Dependent variable: validity of assessment***

What instruments were used to assess the dependent variables (outcome) as explained in the information excel sheet. The answer “yes” can be filled in when validity of all instruments is judged as good. When assessing validity of an instrument, both validity and reliability (inter-rater or test-retest) need to be judged.

Blind assessment, see criteria #6.

When assessments of some outcomes are valid and assessments of others are not, the comment field can be used to specify.

1. ***Was exposure unknown to assessor?***

In theory, someone rating the outcome can rate a little worse in the exposed simply because he thinks the exposure is associated with worse. He might not even be aware of this. This bias can be prevented by asking an independent outcome assessor that does not know the exposure (blind outcome assessor).

1. ***Were subjects excluded when outcome was present at baseline?***

A cohort study always starts with healthy subjects; subjects are “at risk” for the disease. If not, this can lead to biased results.

1. ***Is follow-up sufficiently long to assess the outcome?***

Every research question has its minimum follow up period. Before rating the OSQE, the rater should define this minimum. All studies shorter than this minimum might not show the hypothesized results. This also means that exposure is assessed prior to assessment of the outcome. (Joanna Briggs Institute, 2017, item 8).

1. ***Is the outcome assessed continuously?***

In many cohort studies, data are collected at baseline and at one or more follow-up assessments, while there is no information obtained between the assessments. Thus, when both recovery and relapse occur between two assessments, this remains unknown. Illnesses with a short duration can also easily be missed. Of course, those studies try to overcome this problem by enquiring the period between two assessments. However, then nuances can get lost and there can be recall bias. An outcome such as death is irreversible and thus cannot be missed. When time to event is part of the research question, even in these outcomes information between two assessments is needed. The OSQE gives a star for “outcome is not assessed continuously, but variation in outcome between assessment is not possible (e.g. death)”, but raters can decide that for their research question this is not worth a star.

1. ***Does loss to follow-up likely introduce bias?***

The higher the loss to follow-up the lower the study quality and the higher the risk of bias. When non-response is selective this is even worse. Thus, when both subjects with higher levels of exposure and subjects with different outcomes drop out more often. For example, when subjects with both low socioeconomic status and lung cancer more often drop out, socioeconomic status would seem to protect against lung cancer, while in real there is no association (hypothetical example). The rater can assume selective drop-out except when there is clear evidence that it is non-selective.

A clear cut-off point between high and low loss-to-follow up for all possible research questions cannot be given. The rater is free to change the cut-off of 10% provided in the default version of the information sheet.

1. ***Did the authors use methods to properly deal with missing data (incl loss to follow-up)?***

It is always possible that data collection is not complete. For example, when subjects do not show up for an assessment, when an instrument did not provide valid results, but also loss to follow-up leads to missing data. In the rare situation that data are "missing completely at random", results would be exactly the same whether or not these missing data were included. More frequently, a few variables can be identified predicting missingness, while other variables are similar in subjects with complete data as in subjects with missings (missing at random). In this case, missings can be imputed based on these few variables. When imputations are repeated multiple times (e.g. 1000) results without missing data can be estimated. It is also possible that data are not missing at random. Then imputation is useless, both before and after imputations, results are invalid.

NOTE: In the ***OSQE cross-sectional***, this item is optional and, thus, is moved to the end of the list. Missing data do not originate from loss-to-follow-up when there is no follow-up. However, there can be other reasons for missing information (e.g. data from incomplete medical files). For this reason, the rater has to decide whether or not to score this item (see information sheet).

1. ***Conflict of interest (e.g. funding by pharmacological industry or researcher has an affiliation with the pharmaceutical industry).***

Mainly in medication studies, funding by the pharmacological industry who sells the drug can imply that this industry also had impact on the results. They can prevent publishing results that do not evidence effectiveness of the drug.

1. ***Does the statistical analysis control for the relevant confounders?***

The rater defines which confounders are relevant on the information sheet. The original study needs to effectively control for hypothesized confounders. The sentence that there were no statistically significant differences is not sufficient. Differences at baseline may not be statistically significant because of a lack of power. In that case the variable can still be a confounder.

As dependent and main independent variables, confounders should also be assessed validly. While a variable such as gender is usually valid. Other variables may not be. When assessment is not valid, control for confounders may not be adequate and the item does not obtain a star.

1. ***Did the reporting of the results follow a protocol? In other words, were only a priory intended analyses reported? As opposed to cherry picking***

It is obvious that a researcher should have a hypothesis before he starts writing a paper. Taking a data set and analyzing until nice results are found or analyzing multiple instruments assessing the same construct and only reporting the one that shows a statistically significant association and omitting the other results is not scientific integer (Mayo-Wilson et al., 2017). It is included in the present criteria list because it results in bias towards publishing positive results.

The NOS did not include this item, Strobe only asked whether the authors had a protocol. ROBINS-I and D&B did. The item in D&B asks whether analyses that were not planned at the outset of the study were clearly indicated (data dredging, Downs&Black 1998). ROBINS-I asks for selective reporting, specifying selection from multiple outcome measurements, multiple analyses of the same association, multiple subgroups. So, the question is whether hypotheses presented in the introduction correspond with reported results.

1. ***Are effect modifiers analyzed, correctly?***

Optional.

First the rater has to specify whether there is any hypothesis for effect modification. The answer can be provided in the extra-information excel sheet. When there are no reasons to expect effect modification, this item is not applicable and can be skipped. When effect modification is likely, the authors of the original study should have analyzed this effect modification resulting in two or more test statistics (e.g. regression coefficients) depending on the value of the interacting variables.

1. ***Is sample size sufficient, looking at calculations/explanation provided by the authors?***

Optional.

This question only needs to be answered when the rater does not intend to pool the results, i.e. when performing systematic reviews without meta-analysis or when making a CAT. When pooling small studies get less weight than larger studies. When results of the individual studies are interpreted, the rater also has to give less weight to results of small studies.

***Items I – IV should be self-evident and no extra explanation is provided.***

*B Case-control studies*

*Note that all studies that include cases and controls and that have case-control status as the outcome are case-control studies. Some case-control studies are retrospective making use of registrations or enquiring about the past. Others are cross-sectional. The criteria list is suited for all.*

1. ***Is the sample optimal for both internal validity and representativeness?***

Criterion 1 aims to judge the correct balance between internal and representativeness (external validity). Per definition, an increase in internal validity leads to a decrease in external validity: internal validity is highest in a homogeneous study population with multiple in- and exclusion criteria. External validity is highest when all subjects from a study population are included in the study. So, the more in- and exclusion criteria are formulated the higher the internal validity at the expense of the external validity. For this reason, both items a and item b need to be optimal in order to gain a star.

The rater needs to define what is optimal balance per research question. Thus, a question in the information sheet asks for this balance.

Items c and d further explore representativeness and also need to be optimal. Thus, while on other items a yes on one of the sub-items is sufficient for a star, on this item a star is only given when a to d all are scored yes.

Item c and d: Optimal representativeness means that all cases of a population are included or a random sample of them. To know that all patients from a hospital department over a period are selected, words such as “consecutive” or “all” are informative. This also means that selection is not associated with either exposure or outcome. Controls are often matched with patients. So, in case-control studies it is often mainly representativeness of the cases that matters.

When items a to c point at a representative sample, non-response/refusal can still jeopardize representativeness; mainly when non-response rate is high or selective. Item 1d addresses participation / non-response bias, i.e. when non-participating individuals differ from participating individuals with regard to the assessed independent (exposure) and/or dependent variables (outcome). For example, if one selects a sample of 100 nurses and sent them a survey about their workload. The nurses with a high workload may not complete the survey because they have not enough time to complete it. This selective participation can affect the external validity of the study results. Therefore, it is of importance to evaluate whether there are reasons for individuals to not participate in the study.

Favorable answers:

1a: optimal internal validity

1b: optimal external validity

1c: transparent selection process and representative sample

1d: no reasons for refusal that point at non-representative data

1. ***Is the data collected in one population or are cases and controls selected in different populations?***

Sometimes cases and controls are derived from different populations. For example, when cases are selected from a hospital population, the question is where to find general population controls. How to define the population of individuals who would come to specifically this hospital when they would have had this particular disease? Pragmatically healthy subjects can be selected from a different population. Differences between the populations can lead to biased results. A study obtains a star when all subjects are collected from the same population. When a study oversamples a subgroup within a population to include in either exposed or unexposed this also need to be scored as c (2 or more cohorts from different populations, no star).

On the contrary, in an attempt to obtain controls from the same population subjects from another department of the hospital can serve as controls. However, this leads to a selection within the controls that is unwanted. For example, when studying lung-cancer or psychosis, subjects who are in the hospital with a broken leg can serve as controls. It is not surprising that sports than seems a protective factor against lung-cancer or psychosis in the cases. So, despite controls do stem from the same population, studies with such a design do not receive a star on this item.

Note that cases from the same source population but from a different time period should be scored as NOT from the same source population (Joanna Briggs Institute, 2017).

Favorable answer a or b.

1. ***Independent variable: validity of assessment***

What instruments were used to assess the independent variables (exposure) as explained in the information excel sheet? The answer “yes” can be filled in when validity of all instruments is judged as good. When assessments of some exposures are valid and assessments of others are not, the comment field can be used to specify.

In addition, validity of assessment reduces when there is recall bias. This should be taken into account when scoring this item. Variables such as gender and prospectively collected data from medical files are far less prone to recall bias than dietary questionnaires and variables of which the responder knows it was a risk for the disease he now has (patients with lung cancer better remember that they smoked in the past, while controls forget). This last category of exposure assessments is also prone to observer bias; the assessor could score differently for cases and controls. In that case the rater could specify blinding of case-status as a condition for a star on the information sheet.

Recall bias can be notorious in case-control studies (Rothman and Lash, 2018). It is not a separate item in the OSQE, but part of question 3 (validity of assessment independent variable). In most case-control studies, questionnaires enquire current outcome as well as exposure in the past. Recall bias can be different between cases and controls, because of case-status (e.g. subjects with lung cancer blame asbestos exposure at work, while healthy controls forgot this exposure). Only when objective data from registries is available, or when variables do not change over time (e.g. gender, genotype) recall bias is no problem. When recall bias is likely, item 3 does not obtain a star.

1. ***Presence.***

When subjects are not compliant to medication in an RCT, exposure is not as high as intended. Analogous with that, exposure in observational studies can also differ. For example, childhood exposure to air pollution is lower when the child did not play outside. It depends on the research question how likely differences in quantity of exposure are.

Favorable answer: optimal exposure; 100% compliance; good fidelity; risk factor 100% present.

1. ***Same method of assessment independent variable in cases and controls?***

Because cases and controls are selected separately, data collection can also take place separately. In addition, authors may choose to reduce burden in healthy controls by limiting the number of questions. Did the authors use the same instruments in both cases and controls to assess data needed for the research question of the systematic review?

1. ***Dependent variable: validity of assessment (In other words: Is case definition adequate?)***

In a case-control study, cases and controls are selected even before the data collection.

When assessing validity of an instrument, both validity and reliability (inter-rater or test-retest) need to be judged. When instruments used for case definition is different between cases and controls, item 6 can never obtain a star.

1. ***Controls have no history of disease***

As all subjects in a cohort study (item 7 OSQE cohort), controls should be at risk for the disease. Thus, controls who have the disease should be excluded.

In the rare situation that case-status is not an illness, the rater can replace disease by “outcome of interest”.

1. ***Is follow-up sufficiently long to assess the outcome?***

Every research question has its minimum follow up period. Before rating the OSQE, the rater should define this minimum. All case-control studies enquiring a period shorter ago than this minimum might not show the hypothesized results.

1. ***Does non-response likely introduce bias?***

The higher the non-response, the higher the risk of bias. When there is non-response, it is often selective (also see item 10).

1. ***Is non-response similar in cases and in controls?***

Because healthy population controls are not interested in the disease, the risk is that response is lower. In theory, there can still be selective non-response when non-response is similar between cases and controls (depending on exposure status), but similar non-response does decrease the risk (Vandenbroucke et al., 2007).

1. ***Did the authors use methods to properly deal with missing data (incl loss to follow-up)?***

It is always possible that data collection is not complete. For example, when subjects do not show up for an assessment, when an instrument did not provide valid results, but also loss to follow-up leads to missing data. In the rare situation that data are "missing completely at random", results would be exactly the same whether or not these missing data were included. More frequently, a few variables can be identified predicting missingness, while other variables are similar in subjects with complete data as in subjects with missings (missing at random). In this case, missings can be imputed based on these few variables. When imputations are repeated multiple times (e.g. 1000) results without missing data can be estimated. It is also possible that data are not missing at random. Then imputation is useless, both before and after imputations, results are invalid.

1. ***Conflict of interest (e.g. funding by pharmacological industry or researcher has an affiliation with the pharmaceutical industry).***

Mainly in medication studies, funding by the pharmacological industry who sells the drug can imply that this industry also had impact on the results. They can prevent publishing results that do not evidence effectiveness of the drug.

1. ***Does the statistical analysis control for the relevant confounders?***

The rater defines which confounders are relevant on the information sheet. Matching controls with cases, as is often done in case-control studies, is also a method to decrease confounding. It is most methodologically correct when even matching variables are controlled for in the analysis, because otherwise new confounding is introduced. Thus, only when authors control for the matching variables in the analysis the article receives a star.

1. ***Did the study follow a protocol and were only a priory reasoned results reported? As opposed to cherry picking.***

It is obvious that a researcher should have a hypothesis before he starts writing a paper. Taking a data set and analyzing until nice results are found or analyzing multiple instruments assessing the same construct and only reporting the one that shows a statistically significant association and omitting the other results is not scientific integer (Mayo-Wilson et al., 2017). It is included in the present criteria list because it results in bias publishing positive results.

The NOS did not include this item, Strobe only asked whether the authors had a protocol. ROBINS-I and D&B did. The item in D&B asks whether analyses that were not planned at the outset of the study were clearly indicated (data dredging, Downs&Black, 1998). ROBINS-I asks for selective reporting, specifying selection from multiple outcome measurements, multiple analyses of the same association, multiple subgroups. So, the question is whether hypotheses presented in the introduction correspond with reported results.

1. ***Are effect modifiers analyzed, correctly?***

Optional

First the rater has to specify whether there is any hypothesis for effect modification. The answer can be provided in the extra-information excel sheet. When there are no reasons to expect effect modification, this item is not applicable and can be skipped. When effect modification is likely, the authors of the original study should have analyzed this effect modification resulting in two or more test statistics (e.g. regression coefficients) depending on the value of the interacting variables.

1. ***Is sample size sufficient, looking at calculations/explanation provided by the authors?***

Optional

This question only needs to be answered when the rater does not intend to pool the results, i.e. when performing systematic reviews without meta-analysis or when making a CAT. When pooling small studies get less weight than larger studies. When results of the individual studies are interpreted, the rater also has to give less weight to results of small studies.

***Items I – IV should be self-evident***

Additional reference

39. E. Mayo-Wilson, N. Fusco, T. Li, H. Hong, J.K. Canner, K. Dickersin, and M. investigators, Multiple outcomes and analyses in clinical trials create challenges for interpretation and research synthesis. J Clin Epidemiol (2017) 86: 39-50.
